# Emotional and Behavioral Disorders in Pediatric Cancer Patients

**Published:** 2020

**Authors:** Ghazal ZAHED, Fatemeh KOOHI

**Affiliations:** 1 Pediatric Congenital Hematologic Disorders, Research Center, Research Institute for Children’s Health, Shahid Beheshti University of Medical Sciences, Tehran, Iran; 2 Student Research Committee, Department of Epidemiology, School of Public Health and Safety, Shahid Beheshti University of Medical Sciences, Tehran, Iran.

**Keywords:** Psychiatric disorders, Pediatric patients, Malignancies, Emotional and behavioral disorders

## Abstract

**Objectives:**

Childhood malignancies raise a range of medical, psychological and social concerns. Identifying psychiatric disorders along with providing mental health services to prevent the emergence and aggravation of mental health problems in children seems necessary in pediatric hospitals. We aimed to find out the frequency of probable emotional and behavioral disorders among children and adolescents with malignancy.

**Materials & Methods:**

This was a cross-sectional study conducted at the Hematology-Oncology Ward of Mofid Hospital, Tehran, Iran, during 2017-2018. Emotional and behavioral disorders were assessed in 399 pediatric cancer patients aged 5 to 12 years using the Parent Checklist of CSI-4.

**Results:**

Overall, 89.2% of the samples met the diagnostic criteria for at least one disorder. The most prevalent psychiatric disorders were specific phobia (57%), enuresis (41.9%), obsessive-compulsive disorder (45.6%) and separation anxiety disorder (30.3%). Our results did not show any significant relationship between gender or disease type and the prevalence of psychiatric disorders.

**Conclusion:**

The prevalence of emotional and behavioral disorders in pediatric cancer patients admitted to children’s hospitals is common. These disorders affect the treatment and quality of life of these patients. Therefore, our findings may guide parents, nurses and clinicians to become more cognizant of the identification and management of these disorders.

## Introduction

Cancer is one of the most common diseases in childhood and adolescence (1). The Childhood Cancer Registry of Piedmont (CCRP) reported increasing incidence trends during the period 1967–2011, specifically for leukemia, lymphoma, central nervous system (CNS) tumors and neuroblastoma (2). With the development of new medical treatments and technologies, survival rate of children with cancer has increased (3). However, childhood cancer has a unpleasant impact on patients and their families and it remains a challenge for public health (4). 

Several studies have suggested the increased risk of psychiatric problems in children with medical illnesses (5-9). In addition, evidence demonstrates that there is a bidirectional association between psychiatric disorders and many medical illnesses such that psychiatric disorders might be a causal factor in different illnesses or be resulted of them or they might affect the course of medical illnesses (9). 

In general, hospitalization is a stressful experience for children, and many studies have concluded that children are afraid of illness and hospitalization. Hospitalized children have reported concerns about pain, amputation, lack of mobility, separation from significant people in life, loss of control and confusion (10-13).

The prevalence of anxiety and depressive disorders in children and adolescents with chronic diseases varies from 17% in the society to 33% in clinical samples (7, 8, 14-16).A review of the literature has shown that the incidence and prevalence of emotional and behavioral problems in children and adolescents is on the rise (17). In a study, it was indicated that 33.3% of children with acute lymphoblastic leukemia had emotional disorders (18). In another study, 53% of children with neoplasms were suffering from some emotional and behavioral disorders (3). 

With this background in mind, identifying psychological disorders along with the provision of mental health services in order to prevent the emergence and aggravation of mental health problems of in children seems necessary in pediatric hospitals. 

Most studies on psychiatric disorders have involved children in the community or those with other diseases, which are probably not representative of children with malignancy visiting secondary care, like a pediatric hematology-oncology clinic. Therefore, we aimed to find out the frequency of probable emotional and behavioral disorders among children and adolescents with malignancy and to examine the relationship between the relevant variables and psychiatric disorders among them.

## Materials & Methods

**Participants and study design**

A total of 399 children and adolescents (age: 5 to 12 years) diagnosed with a malignancy were included in this cross-sectional study. Recruitment involved obtaining consent from all the parents/guardians who were accompanying a child with malignancy admitted to the Hematology-Oncology Ward in the Mofid Children’s Hospital in Iran. All the included patients had been diagnosed with malignancy by a pediatric oncologist.

**Procedure**

All the cancer patients aged 5 to 12 years were approached for inclusion in the study. Face-to-face interview was used for data collection. After explaining the study objectives, consent was obtained from the parents, and the hospital’s psychologist administered the study measure to all the parents. The study was approved by the Research Center for Pediatric Congenital Hematologic Disorders in Mofid Children’s Hospital.

**Measure**

*Child Symptom Inventory-4: *CSI-4 comprises of two rating scales, one completed by teachers and one by parents. This scale was developed by Gadow and Sprafkin (1994) to screen 5 to 12-year-old children for symptoms of the common childhood psychiatric disorders based on DSM-IV diagnostic criteria. The Parent Checklist contains 97 items that screen for 17 emotional and behavioral disorders, while the Teacher Checklist contains 77 items that screen for 13 disorders. The assessed disorders include Attention Deficit/ Hyperactivity Disorder (AD/HD), Dysthymia Disorder, Oppositional Defiant Disorder (ODD), Asperger's Syndrome, Conduct Disorder, Pervasive Developmental Disorder, Autistic Disorder, Separation Anxiety Disorder, Schizophrenia, Generalized Anxiety Disorder, Obsessive-Compulsive Disorder, Social Phobia, Posttraumatic Stress Disorder, Specific Phobia, Major Depressive Disorder, Motor Tics Disorder and Vocal Tics Disorder. 

The parent or teacher rates each item based on a 4-point Likert scale, indicating how often the symptom is observed in the child being evaluated. The CSI-4 can be scored for Symptom Count Scores (diagnostic model), which use scores of zero (never/sometimes) or one (often/very often), and Symptom Severity (normative data model) scores, which use scores of 0 (never), 1 (sometimes), 2 (often), or 3 (very often), or Symptom Severity scores (19, 20). Numerous studies performed in Iran have indicated that the CSI-4 has satisfactory internal consistency (Cronbach’s a) and reliability (21-23). 

***Statistical analysis***

After calculating symptom count scores for each disorder in the CSI-4 Parent Checklist, the prevalence of emotional and behavioral disorders was calculated among the children and adolescents with malignancy. Chi-squared test and univariate and multivariate logistic regression were used to determine the relationship between these disorders and gender, age at the time of study, age at the time of diagnosis and duration of treatment. All the data analyses were performed using Stata version 14 at 95% sig-value.

## Results

**Demographic characteristics**

Overall, 399 pediatric cancer patients were included. The mean age of the patients was 8.2 years (SD = 3.9, range 5 to 12. Approximately half of the patients were male (51.4%). Almost one-third of the patients (31.3%) had leukemia and 24.8% had neuroblastoma. The clinical and demographic characteristics of the participants are presented in Table 1.

**Table 1:** Clinical and demographic characteristic of the patients (N=399)

**Variables**	
Age (mean ± SD)	8.2 ± 3.9
**Gender (%)**	
Boy	205 (51.4)
Girl	194 (48.6)
**Disease type (%)**	
Leukemia	125 (31.3)
Neuroblastoma	99 (24.8)
Wilms tumor	75 (18.8)
Lymphoma	51 (12.8)
Sarcoma	49 (12.3)
Age at the time of diagnosis (mean ± SD)	4.2 ± 2.8
Duration of treatment (mean ± SD)	4±3

**The prevalence of emotional and behavioral disorders**

Emotional and behavioral disorders were common among children and adolescents with malignancy admitted to the Hematology-Oncology Ward in Mofid Children’s Hospital (Table 2). In all, 356 (89.2%) of the samples met the diagnostic criteria for at least one disorder. Based on the results, the most prevalent probable diagnosis was special phobia disorder that was prevalent among 57% of the patients. In addition, 45.6% met the diagnostic criteria for the enuresis disorder, 41.9% had obsessive-compulsive disorder and 30.3% had separation anxiety disorder. 

The results suggested that the prevalence of emotional and behavioral disorders was not associated with gender or disease type, although there was a significant relationship between gender and the major depressive disorder (tables 2 and 3). In addition, there was no association between emotional and behavioral disorders and age at the time of study, age at the time of diagnosis and duration of treatment (Table 4).

**Table 2:** Prevalence of the emotional and behavioral disorders in the malignancy patients aged 5-12, by sex (N=399)

**Disorder type**	**Total**	**Boys**	**Girls**	***P*** -value*
	**N (%)**	**N (%)**	**N (%)**	
Attention Deficit Hyperactivity Disorder	112 (28)	56 (14)	56 (14)	0.731
Oppositional Defiant Disorder	77 (19.3)	40 (19.5)	37 (19)	0.911
Conduct Disorder	92 (23.1)	43 (21)	49 (25.3)	0.310
Generalized Anxiety Disorder	30 (7.5)	18 (8.8)	12 (6.2)	0.326
Specific Phobia	228 (57)	115 (56.1)	113 (58.2)	0.664
Obsessive-Compulsive Disorder	167 (41.9)	85 (20.8)	84 (21.1)	0.569
Posttraumatic Stress Disorder	45 (11.3)	25 (12.2)	20 (10.3)	0.552
Motor/Vocal Tics	75 (18.8)	41 (10.3)	34 (8.5)	0.527
Major Depressive Disorder	61 (15.3)	24 (11.7)	37 (19.1)	0.041^a^
Dysthymic Disorder	109 (27.3)	55 (26.8)	54 (27.8)	0.822
Autistic/Asperger's Disorders	45 (11.3)	27 (6.8)	18 (4.5)	0.219
Social Phobia	0	0	0	0
Separation Anxiety Disorder	121 (30.3)	61 (29.8)	60 (30.9)	0.799
Enuresis	182 (45.6)	96 (46.8)	86 (44.3)	0.616
Encopresis	16 (4)	8 (3.9)	8 (4.1)	0.910

*Chi-squared test (x^2^)

a p< 0.05, the χ² test was not significant at the 5% level.

**Table 3:** Prevalence of the emotional and behavioral disorders in the malignancy patients aged 5-12, by disease type (N=399)

**Disorder type**	**Leukemia**	**Neuroblastoma**	**Wilms tumor**	**Lymphoma**	**Sarcoma**	***P*** -value*
	**N (%)**	**N (%)**	**N (%)**	**N (%)**	**N (%)**	
**Attention Deficit Hyperactivity Disorder**	34 (8.5)	29 (7.3)	21 (5.3)	14 (3.5)	14 (3.5)	0.998
**Oppositional Defiant Disorder**	22 (5.5)	19 (4.8)	16 (4.0)	11 (2.8)	9 (2.3)	0.960
**Conduct Disorder**	30 (7.5)	24 (6.0)	16 (4.0)	12 (3.0)	10 (2.5)	0.977
**Generalized Anxiety Disorder**	8 (2.0)	8 (2.0)	6 (1.5)	4 (1.0)	4 (1.0)	0.980^a^
**Specific Phobia**	72 (18.1)	57 (14.3)	42 (10.5)	29 (7.8)	28 (7.0)	1.000
**Obsessive-Compulsive Disorder**	53 (13.3)	42 (10.5)	31 (7.8)	21 (5.3)	20 (5.0)	0.999
**Posttraumatic Stress Disorder**	13 (3.3)	12 (3.0)	9 (2.3)	5 (1.3)	6 (1.5)	0.985
**Motor/Vocal Tics**	23 (5.8)	20 (5.0)	14 (3.5)	10 (2.5)	8 (2.0)	0.986
**Major Depressive Disorder **	96 (24.1)	77 (19.3)	57 (14.3)	39 (9.8)	38 (9.5)	0.999
**Dysthymic Disorder**	34 (8.5)	28 (7.0)	19 (4.8)	14 (3.5)	14 (3.5)	0.994
**Autistic/Asperger's Disorders**	15 (3.8)	12 (3.0)	8 (2.0)	5 (1.3)	5 (1.3)	0.988
**Social Phobia**	0	0	0	0	0	-
**Separation Anxiety Disorder**	36 (9.0)	32 (8.0)	23 (5.8)	15 (3.8)	15 (3.8)	0.986
**Enuresis**	56 (14.0)	45 (11.3)	35 (8.8)	24 (6.0)	22 (5.5)	0.998
**Encopresis**	5 (1.3)	4 (1.0)	3 (0.75)	2 (0.50)	2 (0.50)	1.000^a^

*Chi-squared test (x^2^)

^a^ Fisher's exact test

**Table 4:** Association between emotional and behavioral disorders and continues variables in the malignancy patients aged 5-12 (N=399)

Disorder type	**Age at the time of study**	**Age at the time of diagnosis**	**Duration of treatment**
	OR (CI 95%)*	OR (CI 95%)*	OR (CI 95%)*
Attention Deficit Hyperactivity Disorder	1.14 (0.49-2.7)	0.92 (0.39-2.17)	0.89 (0.37-2.14)
Oppositional Defiant Disorder	0.89 (0.33-2.35)	1.01 (0.93-1.1)	1.01 (0.93-1.1)
Conduct Disorder	0.88 (0.35-2.16)	1.01 (0.94-1.09)	1.01 (0.94-1.09)
Generalized Anxiety Disorder	1.5 (.034-6.9)	0.96 (0.85-1.09)	0.96 (0.85-1.09)
Specific Phobia	0.92 (0.42-2.0)	1.0 (0.94-1.07)	1.0 (0.94-1.07)
Obsessive-Compulsive Disorder	0.95 (0.44-2.07)	0.99 (0.94-1.07)	0.99 (0.94-1.07)
Posttraumatic Stress Disorder	1.6 (0.47-5.59)	0.95 (0.86-1.06)	0.95 (0.86-1.06)
Motor/Vocal Tics	0.72 (0.27-1.93)	1.24 (0.46-3.3)	1.31 (0.48-3.6)
Major Depressive Disorder	2.4 (1.0-5.9)	0.93 (0.86-1.0)	0.93 (0.86-1.0)
Dysthymic Disorder	2.5 (0.93-6.6)	0.93 (0.85-1.0)	0.93 (0.86-1.01)
Autistic/Asperger's Disorders	1.5 (0.45-5.0)	0.64 (0.19-2.14)	0.63 (0.19-2.14)
Separation Anxiety Disorder	1.8 (0.75-4.4)	0.95 (0.89-1.03)	0.95 (0.89-1.03)
Enuresis	1.2 (0.57-2.7)	0.98 (0.91-1.04)	0.98 (0.92-1.05)
Encopresis	0.69 (0.13-3.5)	1.05 (0.91-1.20)	1.01 (0.88-1.17)

*logistic regression test, OR: Odds Ratio, CI: Confidence Interval

## Discussion

To our knowledge, this is the ﬁrst study performed in Iran to investigate emotional and behavioral disorders in pediatric cancer patients referred to a children’s hospital. We found that psychiatric morbidities are common in children and adolescents with malignancy admitted to the Hematology-Oncology Ward in Mofid Children’s Hospital. Using the standardized Parent Checklist of CSI-4, we found an overall prevalence of at least one psychiatric disorder of 89.2%. This prevalence is almost three times higher than that among Indian children with acute lymphoblastic leukemia (33.3%) (18), it is also higher than the prevalence of psychiatric disorders in children with neoplasm referred to a pediatric unit in Bangladesh (53.3%) (3).

In addition, in a population-based study, the prevalence of psychiatric disorders in healthy adolescents from Bangladesh was found to be 15.2% (24), indicating that psychiatric problems are more common among children with chronic physical diseases. The high prevalence rate in our study is in line with previous population studies on psychiatric disorders in children with unexplained chronic pain (8) and asthma (7). Also, the findings of a study performed in a pain clinic setting were in line with our results (25). 

In our study, the most prevalent psychiatric disorders among children and adolescents with malignancy were special phobia disorder, enuresis disorder, obsessive-compulsive disorder and separation anxiety disorder. In Bangladesh, hyperkinetic disorder (45.4%), oppositional defiant disorder (36.3%), specific phobia (25%) and generalized anxiety disorder (14.3%) were the most prevalent emotional and behavioral disorders in children and adolescents with neoplasm (3). In a previous study, main depression disorder (MDD), attention deficit hyperactivity disorder (ADHD) and oppositional defiant disorder (ODD) were the most prevalent psychological disorders in diabetic children and adolescents (26).

Our findings did not show any marked gender differences in the prevalence of psychiatric disorders. However, a significant association was found between major depressive disorder and gender, that is, the prevalence of this disorder was higher in girls than boys. This result is inconsistent with the results of similar studies (3, 24). Furthermore, in the present study, there was no difference in the prevalence of emotional and behavioral disorders among patients with different malignancy types. A previous study reported no difference in the overall severity of psychiatric disorders between children with leukemia and children with other chronic diseases like diabetes or asthma (27).

In addition, our findings suggested no association between emotional and behavioral disorders and age at the time of study, age at the time of diagnosis and duration of treatment. The high prevalence of psychiatric disorders in pediatric cancer patients has consequences in clinical practice as well as their adult life (24). Furthermore, our results imply that for children with chronic diseases, especially malignancies, psychiatric work up is necessary. When a child presents with malignancy, it is essential to conduct a careful psychiatric assessment, and evidence-based child psychiatric treatment should be offered if psychiatric disorders are present.

This study has some limitations that should be addressed in future studies. The first one is the lack of a comparison group that did not allow us to compare the prevalence of psychiatric disorders with pediatric patients with another chronic disease or healthy children in the community. The second one is applying a self-report tool which might have resulted in overestimated rates. Therefore, future studies are suggested to include a comparison group and diagnostic methods that are more accurate and sensitive.

**In Conclusion, **The prevalence of emotional and behavioral disorders in pediatric cancer patients is high. The identification and management of psychiatric disorders in children and adolescents with a chronic disease including malignancy would improve treatment and the quality of life. Therefore, these findings may guide parents, nurses and clinicians to become more cognizant of these disorders.
